# Improving prediction of rare species’ distribution from community data

**DOI:** 10.1038/s41598-020-69157-x

**Published:** 2020-07-22

**Authors:** Chongliang Zhang, Yong Chen, Binduo Xu, Ying Xue, Yiping Ren

**Affiliations:** 1 College of Fisheries, Ocean University of China, 216, Fisheries Hall, 5 Yushan Road, Qingdao, 266003 China; 2 School of Marine Sciences, University of Maine, Libby Hall, Orono, ME 21604469 USA; 3 Field Observation and Research Station of Haizhou Bay Fishery Ecosystem, Ministry of Education, Qingdao, 266003 China; 4 Laboratory for Marine Fisheries Science and Food Production Processes, Pilot National Laboratory for Marine Science and Technology (Qingdao), 1 Wenhai Road, Qingdao, 266237 China

**Keywords:** Biogeography, Community ecology, Ecological modelling

## Abstract

Species distribution models (SDMs) have been increasingly used to predict the geographic distribution of a wide range of organisms; however, relatively fewer research efforts have concentrated on rare species despite their critical roles in biological conservation. The present study tested whether community data may improve modelling rare species by sharing information among common and rare ones. We chose six SDMs that treat community data in different ways, including two traditional single-species models (random forest and artificial neural network) and four joint species distribution models that incorporate species associations implicitly (multivariate random forest and multi-response artificial neural network) or explicitly (hierarchical modelling of species communities and generalized joint attribute model). In addition, we evaluated two approaches of data arrangement, species filtering and conditional prediction, to enhance the selected models. The model predictions were tested using cross validation based on empirical data collected from marine fisheries surveys, and the effects of community data were evaluated by comparing models for six selected rare species. The results demonstrated that the community data improved the predictions of rare species’ distributions to certain extent but might also be unhelpful in some cases. The rare species could be appropriately predicted in terms of occurrence, whereas their abundance tended to be underestimated by most models. Species filtering and conditional predictions substantially benefited the predictive performances of multiple- and single-species models, respectively. We conclude that both the modelling algorithms and community data need to be carefully selected in order to deliver improvement in modelling rare species. The study highlights the opportunity and challenges to improve prediction of rare species’ distribution by making the most of community data.

## Introduction

Species distribution model (SDMs) have been widely used to evaluate ecological niches and to predict geographic distribution of organisms across terrestrial, freshwater, and marine habitats^1–6^. A majority of SDMs have been developed for common and economically important species because of practical incentives, while predictive models are more challengeable for rare species due to methodological difficulties^7–9^. As most species are rare in natural biological communities^10,11^, modeling common species cannot depict the full picture of biodiversity. In addition, rare species, characterized by low occurrence, are particularly vulnerable to environmental changes and human impacts thus deserve special concerns in biological conservation^8,12^. As such, there is a pressing need to predict the distribution of rare species for successful conservation in the practices of designing marine protected areas (MPAs) and identifying priorities for monitoring programs^13^.

Accurate prediction of rare species is not easy. The difficulties come largely from the limits of data, as the observations of rare species are typically sparse in terms of spatial location and temporal frequency^14–16^. The sparse data imply that the number of presence observations is often small compared to the number of influential predictors, resulting in a critical problem of over-fitting in modelling^8,16,17^. Besides, occurrence or abundance of rare species are often vulnerable to sampling errors, which may lead to model misspecification, making it unfeasible to characterize species’ niche space^18^. There are a few studies aiming to address the issue of rarity, e.g., by developing a large number of simple models averaged in an ensemble^8,9^, and generating pseudo-absence from a habitat suitability map^19–21^. In spite of the progress, many issues remain, such as species’ nonlinear responses to environmental variables^2,22^, unobserved/unknown driving forces^23^, imperfect detection^16,24^, among other outstanding difficulties^25^.

With the development of modern statistics, technical advances provide powerful tools to estimate and predict species distributions, for example, machine learning methods and Bayesian hierarchical models are highly flexible to handle complex ecological responses and are promising for data-limited situations^26–28^. Some predictive methods have emerged to account for community information, leading to a new modelling approach known as community-level models^29^ or joint species distribution models (JSDMs)^30–33^.This modelling approach may benefit the prediction of rare species by borrowing strengths from community data^29,34–36^, which include rich information of species correlations resulting from biological interactions or shared environmental gradients^30,37,38^. These factors have essential influences on species distributions thus may improve the predictive powers of species distribution models. That is, models that integrate community data may contribute to solving the ‘rare-species modelling paradox’.

It should be acknowledged that this idea of community modelling is not quite new^39,40^, and some studies have compared the performances between single- and multi-species models^41–43^. However, JSDMs remain underutilized to date^29,44,45^, and there are limited understanding of their advantages and limitations. Although many studies suggest JSDMs may outperform single-SDMs (SSDMs), the advantage is not guaranteed^29^, and JSDMs may lead to biased parameters if some species have responses to the environment very different from others. Therefore, the gains of adopting JSDMs need to be carefully considered.

This study tested the predictive performances of rare species distribution models, focusing on the hypothesis that community data may improve model prediction. We chose a range of SDMs that treat community data in different ways^29^ and compared their performances using cross validation with survey data collected in the coastal water of Yellow sea, China. Both species occurrence and abundance were considered in the evaluation, as studies have concentrated on occurrence data but abundance data are better indicators of extinction risk^42,46^. In addition to comparing modelling algorithms, we evaluated two approaches of data arrangement, species filtering and conditional prediction, to enhance the predictive performances of the chosen models. These approaches were considered from a pragmatic viewpoint, i.e., available data and modelling techniques are often fixed and can be hardly improved in time, and improving model prediction, even to a limited extent, may be the only solution to account for the rare-species challenge. The goal of this study is to improve our ability to predict the spatial distribution of rare species for biological conservation.

## Results

### Variations in predictability

**Figure 1:**
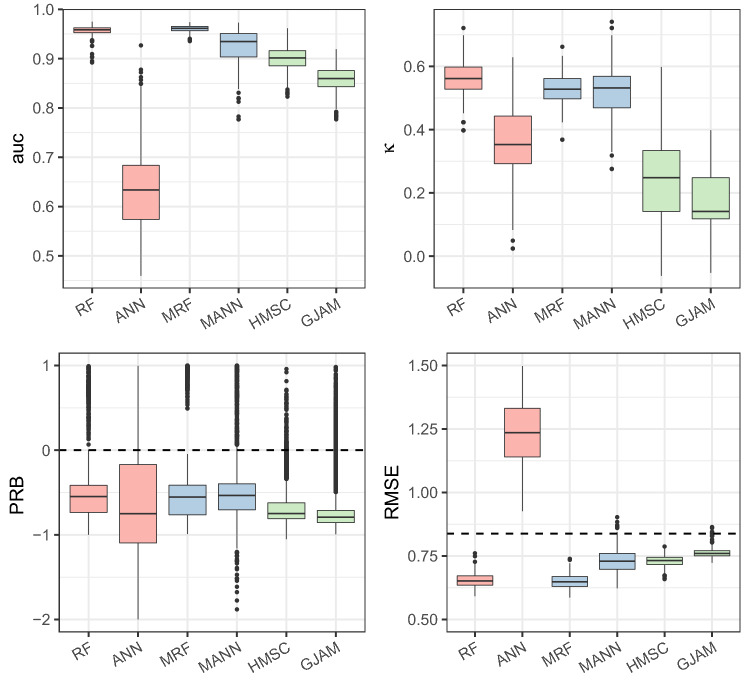
Predictive performances of models on the distribution of Japanese seahorse (*Hippocampus mohnikei*). The prediction of occurrence was evaluated by the area under the curve of receiver operating characteristic (auc) and Cohen’s coefficient (κ), and prediction of abundance was evaluated by partial relative bias of non-zero data (PRB) and root mean square error (RMSE). The dash line in the last plot denotes a baseline of RMSE derived from all-zero predictions.

The tested SSDMs and JSDMs had substantially different predictive abilities. Considering the results of Japanese seahorse (*Hippocampus mohnikei,* Sp4), AUCs (the area under curve of receiver operating characteristic) around 0.9 showed that occurrence of this species could be properly predicted by most models, except artificial neural network (ANN) (Fig. 1). The Cohen’s κ coefficient indicated a similar pattern, whereas hierarchical modelling of species communities (HMSC) and generalized joint attribute model (GJAM) performed worse than those machine learning methods. The results of RMSE (root mean square error) were consistent with AUC, and ANN yielded RMSE larger than that simply assuming the absence of this species over survey areas (dash line). All the models had negative partial relative bias (PRB) on average, implying the tendency of underestimating abundance. The results of other five species showed a similar pattern but the values of performance metrics varied substantially (Supplementary Figure S5). In general, multivariate random forest (MRF) and random forest (RF) showed the best predictive powers for this species, followed by multi-response artificial neural network (MANN).

The divergences in the model performances were compared for other species. In terms of occurrences, MRF provided the best predictions of Sp1 (Brown croaker, *Miichthys miiuy*) and Sp3 (Blackhead seabream, *Acanthopagrus schlegelii*), and RF was optimal for Sp5 (Black scraper, *Erisphex pottii*). HMSC and MANN provided better predictions of Sp2 (Ocellate spot skate, *Raja porosa*) and Sp6 (Bartail flathead, *Platycephalus indicus*) in some measurements (Table 1). The cases of RMSE were complicated, i.e., HMSC and GJAM was the best for Sp1 and Sp2, respectively, RF best for Sp3 and Sp5, and MRF for Sp4 and Sp6. It should be noted that the discrepancies among models were relatively small in terms of the performance metrics, especially between RF and MRF. The predictions of abundance were poor for very rare species, and no model made better predictions than assuming all-zeros for Sp2. In addition, relative performances of the models were not consistent among species. Sp3, Sp4 and Sp6 were more readily predicted than the other species (Table 1). The occurrence of the rarest species in this study, Sp1, could be properly predicted, whereas Sp2 and Sp5 were less well predicted in terms of both occurrence and abundance.

**Table 1:** A summary of model predictive performances for target rare species.

Measures	Models	Sp1	Sp2	Sp3	Sp4	Sp5	Sp6
AUC	RF	0.875	0.644	0.949	0.959	0.800	0.911
	ANN	0.711	0.572	0.628	0.634	0.582	0.633
	MRF	0.893	0.618	0.956	0.962	0.784	0.926
	MANN	0.722	0.576	0.929	0.935	0.751	0.929
	HMSC	0.802	0.670	0.941	0.901	0.765	0.908
	GJAM	0.724	0.640	0.932	0.860	0.688	0.896
κ	RF	0.208	− 0.046	0.486	0.562	0.289	0.616
	ANN	0.134	0.030	0.358	0.353	0.102	0.320
	MRF	0.243	0.163	0.601	0.528	0.205	0.644
	MANN	0.088	0.030	0.486	0.532	0.243	0.634
	HMSC	0.041	− 0.034	0.493	0.248	0.126	0.541
	GJAM	0.046	− 0.034	0.408	0.142	0.069	0.497
RMSE	RF	0.299	0.419	0.377	0.652	0.569	0.588
	ANN	0.652	0.797	0.533	1.259	1.402	1.195
	MRF	0.300	0.416	0.414	0.648	0.577	0.559
	MANN	0.337	0.453	0.389	0.729	0.612	0.587
	HMSC	0.297	0.414	0.384	0.732	0.569	0.611
	GJAM	0.300	0.409	0.393	0.760	0.582	0.628
	Zero	0.300	0.397	0.418	0.838	0.601	0.776

Each cell denotes the average values of the performance measures for a combination of species and models, respectively. Large values of AUC and κ represented high predictive accuracy of species occurrence and small values of RMSE represent high predictive accuracy of species abundance. The row of “Zero” denotes a baseline of RMSE when all predicted values are zeros.

### Species filtering

**Figure 2:**
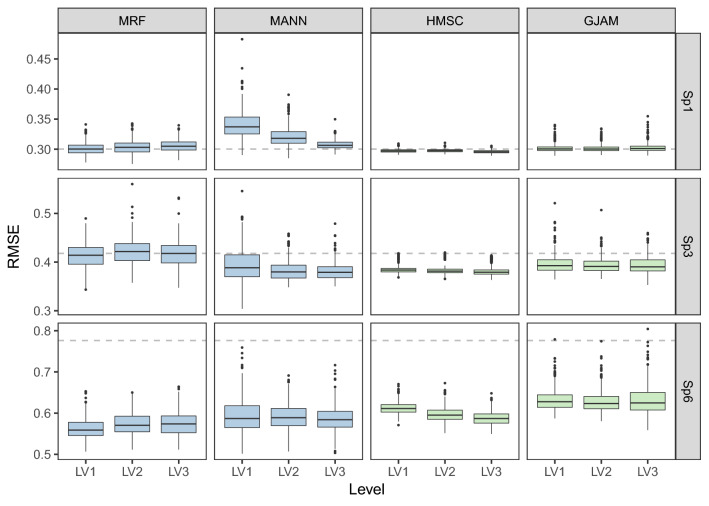
The influences of species filtering on the predictive performance of JSDMs. The levels in the X-axis denoted different thresholds of species correlation for selecting ancillary species (LV1 denoted a large set of species selected and LV3 denoted a small set. Three species are illustrated as examples and the full results are shown in Supplementary Information).

The increasing thresholds of species selection (filtering) led to less but strongly correlated species, which imposed different effects on the four JSDMs (Fig. 2). Among them, MRF tended to be less responsive to the changes of species selection, and the corresponding RMSE increased slightly only for Sp2 and Sp6 in LV3 (levels of species filtering, and LV3 denoted a small set of species selected). On the contrary, the predictions of MANN were substantially improved by reducing the number of species with decreasing RMSE, except for Sp6. HMSC was barely influenced in the cases of Sp1, Sp2 and Sp3 but benefited from specie selection for other species. GJAM also showed less responses to species selection for Sp1, 2, 3, but its performances decreased in terms of the other species. At LV3, MANN and HMSC tended to outperform the other models.

### Conditional predictions

**Figure 3:**
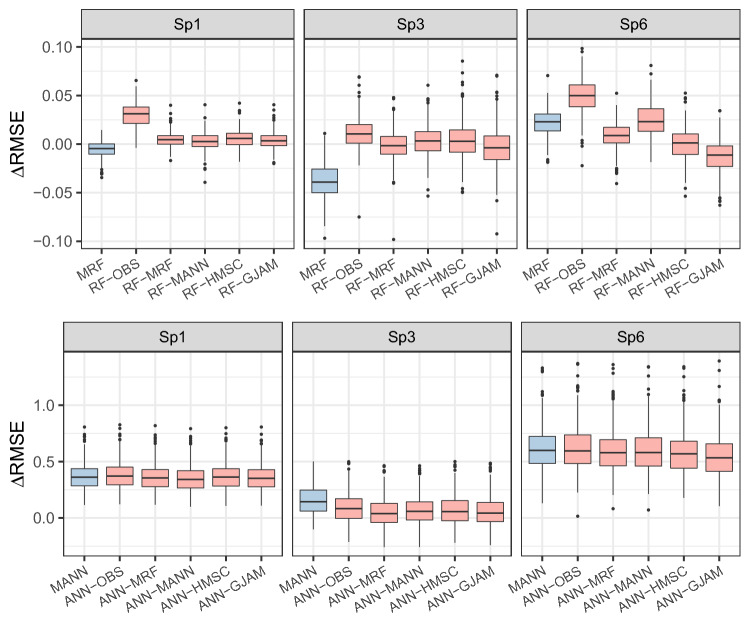
The effects of conditional prediction on improving predictive performances. The ΔRMSE indicates the decreases of RMSE in conditional models compared to that of single-species RF and ANN, respectively. RF-OBS and ANN-OBS denote the predictions conditioning on real observations (survey data), and others are conditioning on the prediction of JSDMs (Three species are illustrated as examples and the full results are shown in Supplementary Information).

Comparing to single-species RF, the predictive accuracy of conditional-RF (using ancillary species as predictive variables) was substantially improved for most species, indicated by the decreases in RMSE (ΔRMSE in Fig. 3). Predictions conditioning on observation data of ancillary species (RF-OBS) showed the most gains of accuracy; meanwhile, comparable improvement could be obtained with the help of JSDMs, i.e., conditional-RF based on JSDMs (using the outputs of JSDMs as predictors) could substantially improve RF, which performed better than MRF in many cases.

Conditional predictions also remarkably improved ANN to the performance similar to or better than MANN (Fig. 3). The degrees of improvement showed small differences between observation-based and model-based conditioning. However, the effects substantially differed among species, largest for Sp5 and Sp6 and least for Sp3.

## Discussion

Given the global awareness of biodiversity loss with climate changes and anthropogenic pressures, it is not surprising that SDMs have been increasingly used in recent years. It is therefore of great concern how reliable the models are in their utility of predicting species distribution^47–49^. Here in this study, we examined the performances of a representative selection of modelling methods for rare species using a typical dataset available in marine fisheries surveys. Our results were generally mixed, that is, most species could be appropriately predicted in terms of occurrence, whereas non-zero abundance tended to be underestimated. Nevertheless, given the rather limited occurrence (mostly less than 10%), such performances were acceptable for rare species in a context of biological conservation. Although the conclusions may depend on specific objectives of studies and characteristics of targeted ecosystems, we highlight the opportunities of community data to address the ‘rare-species modelling paradox’^30,35^.

It is worth noting that this study covers a limited scope of SDMs in a continuous spectrum of complexity, and the potential of existing models may not be fully reflected. In particular, literature have concluded that the predictive abilities of SDMs may vary in different circumstances, depending on the type of organisms, their life-history trait, behavior, prevalence, data quality, spatial resolution and extent, and the impacts of human activities^17,25,50,51^. The target species in this study by no means represent the high diversity of marine organisms. In particular, the so-called ‘rare species’ may also diverge in definition, characterized by geographic range, habitat specificity and local density, and different types of “rarity” may influence predictive models in different ways^16,52,53^. In general, substantial challenges still lie ahead on the road to predicting rare species.

In our evaluation, the six models had divergent performances when evaluated with different objectives, measures and target species. In general, the models using RF algorithms had better predictive ability than ANN- and regression-based models for both occurrence and abundance. The advantage could be largely attributed to the successful control of overfitting by model ensembles and internal cross-validation^54^. On the other hand, ANN easily led to overfitting under the circumstance of sampling errors and environmental noise^55^. Nevertheless, the predictive power was substantially improved in MANN and conditional ANN, implying that the overfitting issue was effectively alleviated by borrowing information from common species. On the other hand, the regression algorithm adopted by HMSC and GJAM implied that they were less flexible to non-linear relationships^30^ and at the same time less vulnerable to overfitting^56^. Whereas, the regression-based JSDMs tended to be ‘conservative” for rare species in terms of PRB. We highlight that model ensemble and internal cross-validation should be considered in the future development of SDMs, and particularly the capacity to account for non-linearity and overfitting for JSDMs^57^.

Considering the overall performances of the SDMs, our evaluations generally find better predictive powers in the category of machine-learning JSDMs and conditional SSDMs, suggesting that community information are useful for the prediction of rare species^3,6^, although the extent of improvement depends on the statistical algorithms adopted. It is well established that such gains could be attributed to the covariations in species distribution, as a result of (dis)similar environmental requirements, biotic interactions such as competition and predation, human impact such as fishing, and other stochastic processes such as observation/sampling errors^29,30,32^. Our results are consistent with this conclusion, i.e., species less correlated with the others (Sp2) tend to be poorly predicted while the well predicted one (Sp3, Sp4 and Sp6) show relative high correlations in the raw data (Supplementary Fig. S2). Meanwhile, it should be noted that SSDMs, specifically RF, may outperform the community models when predicting rare species, implying that community information are not helpful in certain circumstances. This is because the underlying driving forces may be idiosyncratic for the target species and others^29,58^. In this case, the distributional patterns of rare species reflected by the limited data may be concealed by the relatively large amount of data of common species, and increasing species number may make the situation worse for model fitting. Such a result was evident in the species selection processes in MANN and HMSC, both of which tended to have improved predictive powers when the number of species was reduced. On the other hand, MRF showed less responses to species selection because the RF algorithm could effectively suppress predictor species with loose correlations^54^. The declining performance of GJAM might also be attributed to the predicting algorithm, which generated latent variables randomly from a multivariate normal distribution according to species covariance matrices^59^. In this case, a strong correlation matrix might lead to larger prediction of latent variables and increased RMSE for rare species. Our results highlight the critical role of species selection in the implementation of JSDMs especially MANN and HMSC.

This study provides suggestions for the application of SDMs for rare species. First, MRF, conditional RF and HMSC are recommended provided the models properly tuned in structure and input variables. Conditional RF should be most powerful for modelling rare species when the distribution of common species are known in the locations of interest (RF-OBS). These results may contribute to extending the scope of species that can be statistically modelled and facilitating studies of similar backgrounds in the cases of rare species or limited data. In future studies, in addition to the improvement of data quality and quantity, algorithmic development is still in need to address the multiple issues raised by rarity. As no models is likely to be superior in all circumstances, diverse types of SSDMs and JSDMs with different features should be combined to address different situations of biological characteristics, rarity and available data, for which better understanding of potential and shortcoming of the existing models are required. Finally, regarding the challenges far from solved, we highlight the need of research efforts in the field of modelling rare species to deliver successful ecosystem management and biodiversity conservation.

## Methods

### Study area and data

A marine fisheries survey was conducted in the north Yellow Sea, China to collect data. A modified systematic survey design was implemented with a total of 118 sampling stations in 2017 (Supporting information, Supplementary Fig. S1). In each station, an otter trawl which has the net width of 15 m and cod-end mesh size of 20 mm was towed for around 1 h at a speed of nearly 3 knots. Catch data were standardized to the same sampling efforts (trawling speed *time) for modelling. The survey and analysis methods were carried out in accordance with the ethics and guideline of the China law and the experimental protocol is approved by Ethical committee of Ocean University of China.

A total of 145 fish, shrimp and cephalopod species, in addition to benthos, were identified in the survey. As this study concentrated on rare species, only species occurring in less than 15% of the survey stations were selected as target species. As a result, six species with the occurrence frequency ranging from 3 to 12% were selected, including Brown croaker (*Miichthys miiuy,* Sp1, 3.5%), Ocellate spot skate (*Raja porosa,* Sp2, 4.3%), Blackhead seabream (*Acanthopagrus schlegelii,* Sp3, 6.1%), Japanese seahorse (*Hippocampus mohnikei,* Sp4, 8.8%), Black scraper (*Erisphex pottii,* Sp5, 9.6%), and Bartail flathead (*Platycephalus indicus,* Sp6, 12.3%) (Supplementary Table S1 in Supporting Information). In addition, 31 most prevalent species with occurrence frequency ranging from 23 to 87% were used as ancillary species (Supplementary Fig. S2) to help the prediction of target species. Commonly available hydrological variables in marine surveys were measured, including bottom water temperature, salinity, and depth (details are shown in Supplementary Table S2; Supplementary Fig. S3), using a CTD system (XR-420) in the same sampling stations after hauling.

### Predictive models

We selected six SDMs following three approaches in terms of how species associations are utilized. The first modelling approach is single-species distribution models (SSDM), which refer to the traditional methods that exclude community data. Two commonly used models, random forest (RF)^60^ and artificial neural network (ANN)^61^ are adopted. The two models are selected because they are powerful and can automatically deal with non-linear relationships that are prevalent in ecological studies^62,63^. The two models are used as references to evaluate how community information may improve the prediction of rare species distribution.

The second approach includes multivariate random forest (MRF) and multi-response artificial neural network (MANN), which are extensions of RF and ANN to account for multiple response variables, respectively. The former is analog to RF in term of bootstrap resampling but the split function is modified to minimize species compositional similarity within groups^64,65^. The latter MANN shares the same algorithm with ANN whereas its output layer has multiple neurons^66^. The connection coefficients between input and hidden layers affect all species collectively in MANN. Although both MRF and MANN are designed for modelling community data, their algorithms account for the information of species associations implicitly (c.f. the following category).

The third approach accounts for species associations explicitly, including two JSDMs that adopt the Bayesian hierarchical framework. The first is a versatile statistical framework of hierarchical modelling of species communities (HMSC)^32^, which uses latent variables to incorporate information of species associations^32,67^. The other is generalized joint attribute model (GJAM), designed to accommodate multifarious data types flexibly, such as presence-absence, ordinal, continuous, discrete, composition and censored data^59,68^. The model represents species responses using a latent continuous variable, which can be censored to the discrete space of observations.

All the models were implemented on the R platform (version 3.5.1), using packages “randomForest”, “nnet”, “MultivariateRandomForest”, “HMSC”, and “gjam”, respectively. A summary of the models was provided in Table 2, and additional technical details were shown in Supporting Information.

**Table 2:** A summary of predictive models used in this study.

Categories	Models	Full names	How to address species associations	R packages	References
SSDM	RF	Random forest	None	randomForest (v4.6-14)	Breiman^60^
	ANN	Artificial neural network	None	nnet (v7.3-12)	Basheer and Hajmeer^73^
Machine-learning JSDM	MRF	Multivariate random forest	Implicitly incorporated from compositional similarity	MultivariateRandomForest (v1.1.5)	Segal and Xiao^64^
	MANN	Multiresponse artificial neural network	Implicitly incorporated from neuron connections	nnet (v7.3-12)	Olden^66^
Regression-based JSDM	HMSC	Hierarchical Modelling of Species Communities	Explicitly incorporated with latent variables	HMSC (v2.2-0)^a^	Ovaskainen et al.^32^
	GJAM	Generalized Joint Attribute Model	Explicitly incorporated with a covariance matrix	gjam (v2.2.6)	Clark et al.^59^

^a^R codes of HMSC are available on Github (https://github.com/guiblanchet/HMSC), and others are available on CRAN.

### Prediction improvement

We tested two approaches to improving predictions of JSDMs and SSDMs, using species filtering and conditional prediction, respectively. It should be noted that the “improved” models used the same algorithms as above, whereas the variables used for model fitting varied. The first approach followed the concern that community models might not benefit predictions when the response variables were poorly correlated^29^. To avoid the undue influences, we selected ancillary species from the 31 common ones according to their correlations with target species. Three levels of species filtering were considered, level-1 (LV1) included all 31 common species, level-2 (LV2) included two-third species of the highest correlations, and level-3 (LV3) with the first third of the highest correlations. The process of species selection was conducted for each target species, and JSDMs were fitted with target species and their corresponding ancillary species at different levels of thresholds (LV), respectively.

The second approach, conditional prediction, was designed to improve the SSDMs using ancillary species directly as predictive variables^69^. The ancillary species were considered in two scenarios, one that ancillary species were observed in all sampling sites, and the other that they were predicted from JSDMs. Obtained from either way, the information of ancillary species were used in SSDMs as predictive variables. To suppress noise and reduce the number of predictive variables, principal component analyses (PCA) were conducted on ancillary species data prior to model fitting, and only PCs with eigenvalues above one were included in the conditional models^70^.

### Evaluation procedures

A four-fold cross validation procedure was used to evaluate models’ predictive performances. The total data were split into four equal sized subsamples, in which 75% were used for model training and the remaining 25% for testing, iteratively. To avoid potential failures with all-zero training/testing dataset, the nonzero data of rare species were randomly assigned to the four subsamples to ensure that each had equal number of occurrence of target species. Specifically, data splitting was conducted separately for samples with and without target species, and a permutation process was used to assign the survey data to four subsamples.

The predictive performances for species abundance were measured by root mean square error (RMSE) between observations and model predictions, RMSE = $\sqrt{\sum_{i}^{N}{({P}_{i}-{O}_{i})}^{2}/N}$, where P*i* and O*i* were the prediction and observation of abundance in sampling site *i*, respectively (RMSE thus has the same unit as abundance and the unit is omitted in the texts). In addition, we concerned the models’ predictive power for non-zero observations and used partial relative bias (PRB) to measure predictive accuracy in the sampling sites where target species were present, i.e., PRB = $({P}_{p}-{O}_{p})/{O}_{p}$, where O_*p*_ was non-zero observations and P_*p*_ was the prediction in the corresponding sampling site.

Performances on predicting species occurrence were measured by the area under curve (AUC) of receiver operating characteristic and Cohen’s κ coefficient^17^. The former has been commonly used for model evaluation of presence-absence, and the latter is used to indicate the chance-corrected agreement between predictions and observations^71^. A random guess of occurrence leads to 0.5 and zero in AUC and Cohen’s κ, respectively. Additionally, True Skill Statistics^72^ were calculated and shown in the Supporting Information. Given that low detectability of rare species might lead to zero observations, a species-specific threshold, mean abundance in the whole area, was used to determine species occurrence from predicted abundance. Data splitting, model fitting, prediction, and evaluation were conducted for each of the target species, and the processes of cross-validation were repeated 500 times.

## Supplementary information

Supplementary information

## Data Availability

Data and R codes may be available from the Dryad Digital Repository.
