# Supplementary material for: Improving prediction of rare species’ distribution from community data
Source: Sci Rep. 2020 Jul 22;10:12230. doi: 10.1038/s41598-020-69157-x (PMC7376031; doi:10.1038/s41598-020-69157-x)
Supplement: Supplementary file 1 — Supplementary information [file 41598_2020_69157_MOESM1_ESM.docx]

Supporting information for

**Improving prediction of rare species’ distribution from community data**

Chongliang Zhang^1^, Yong Chen^2^, Binduo Xu^1^, Ying Xue^1^, Yiping Ren^1,3,4^

1. College of Fisheries, Ocean University of China. 216, Fisheries Hall, 5 Yushan Road, Qingdao, China, 266003; 2. School of Marine Sciences, University of Maine. 216, Libby Hall, Orono, ME, U.S.A. 04469; 3. Field Observation and Research Station of Haizhou Bay Fishery Ecosystem, Ministry of Education, Qingdao, China, 266003; 4. Laboratory for Marine Fisheries Science and Food Production Processes, Pilot National Laboratory for Marine Science and Technology (Qingdao), Qingdao 266237, China, 1 Wenhai Road, Qingdao, China, 266000.

Supporting information include a summary of community data (Table S1 and Figure S2), a summary of environmental variables (Table S2 and Figure S3), technical details of model specification (Table S3), and supplementary results (Figures S4-8).

Figure S1 Survey area.

Table S1 A list of the target species.

Figure S2 Species correlations in the raw data.

Table S2 A summary of the environmental variables.

Figure S3 Maps of the environmental variables.

Table S3 Technical details of model specifications.

Figure S4 Principal component analyses on model predictions

Figure S5 Performances of SDMs on six target species.

Figure S6 The effects of ancillary species.

Figure S7 True skill statistic (TSS) of model predictions.

Figure S8 The effects of conditional prediction.


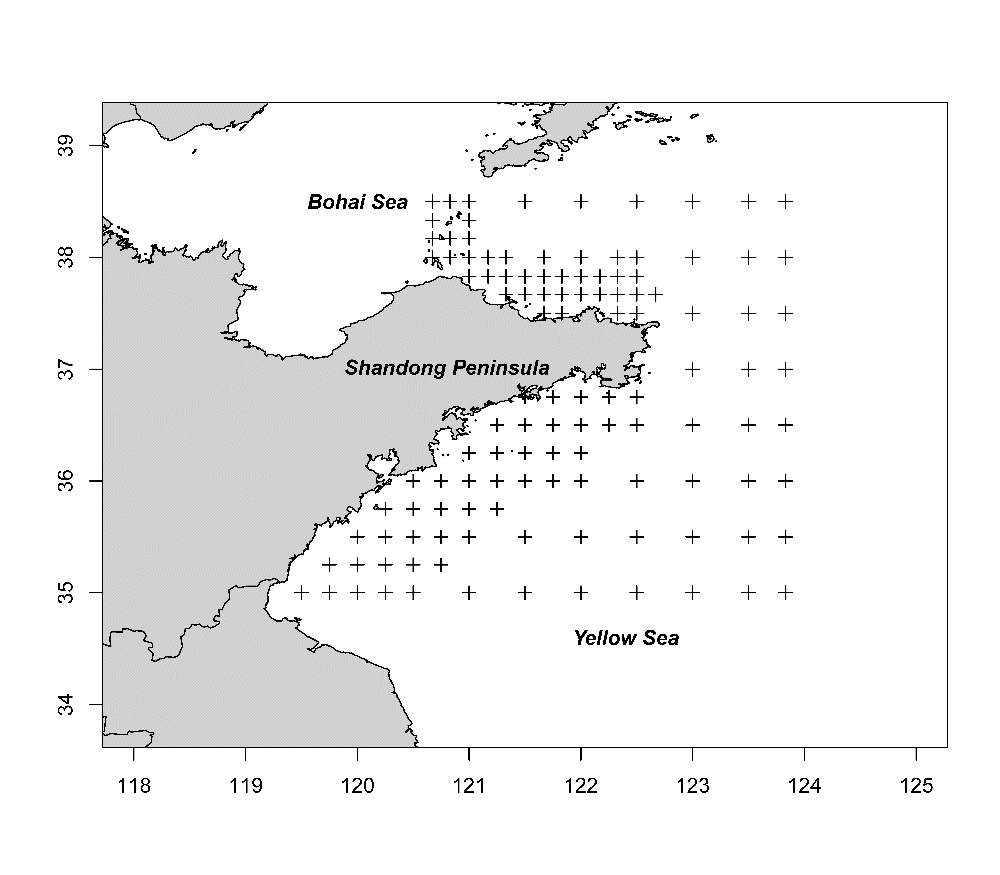
Figure S1 Marine fisheries survey area and sampling stations in Shandong Coastal area. A total of 118 sampling stations (denoted with cross) were investigated in October 2017. It should be noted that survey stations were relatively dense in the coastal area and less so in offshore, considering intense environmental gradients in the former. The map were created using R package “maptools” (version 0.9-5).

Table S1 A list of the target species with their occurrence in the survey data.

| Species | Common names | Scientific names | Occurrence | Total abundance | Fishbase links |
| --- | --- | --- | --- | --- | --- |
| 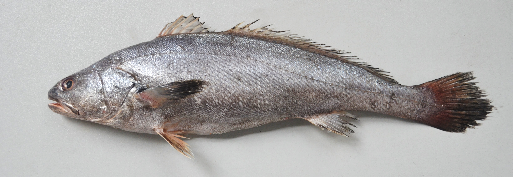 | Brown croaker | *Miichthys miiuy* | 4 | 17 | <http://www.fishbase.org/summary/Miichthys-miiuy.html> |
| 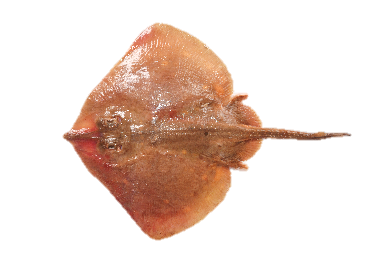 | Ocellate spot skate | *Okamejei kenojei* | 5 | 40 | <http://www.fishbase.org/summary/Okamejei-kenojei.html> |
| 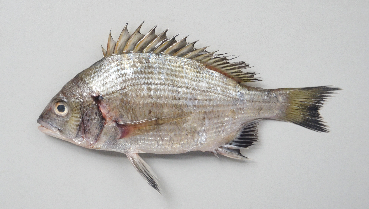 | Blackhead seabream | *Acanthopagrus*  *schlegelii* | 7 | 44 | <http://www.fishbase.org/summary/Acanthopagrus-schlegelii.html> |
| 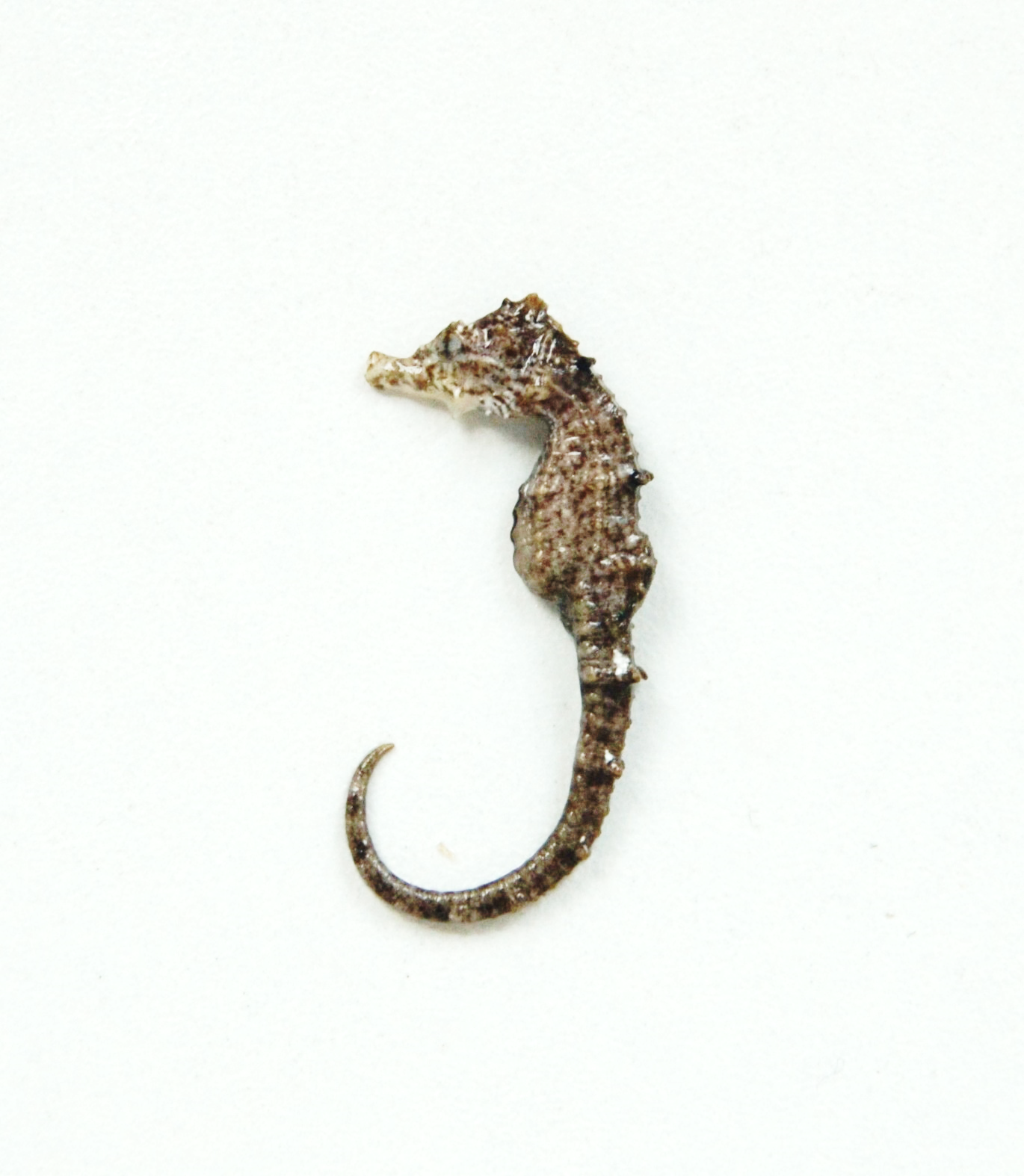 | Japanese seahorse | *Hippocampus mohnikei* | 10 | 184 | <http://www.fishbase.org/summary/Hippocampus-mohnikei.html> |
| 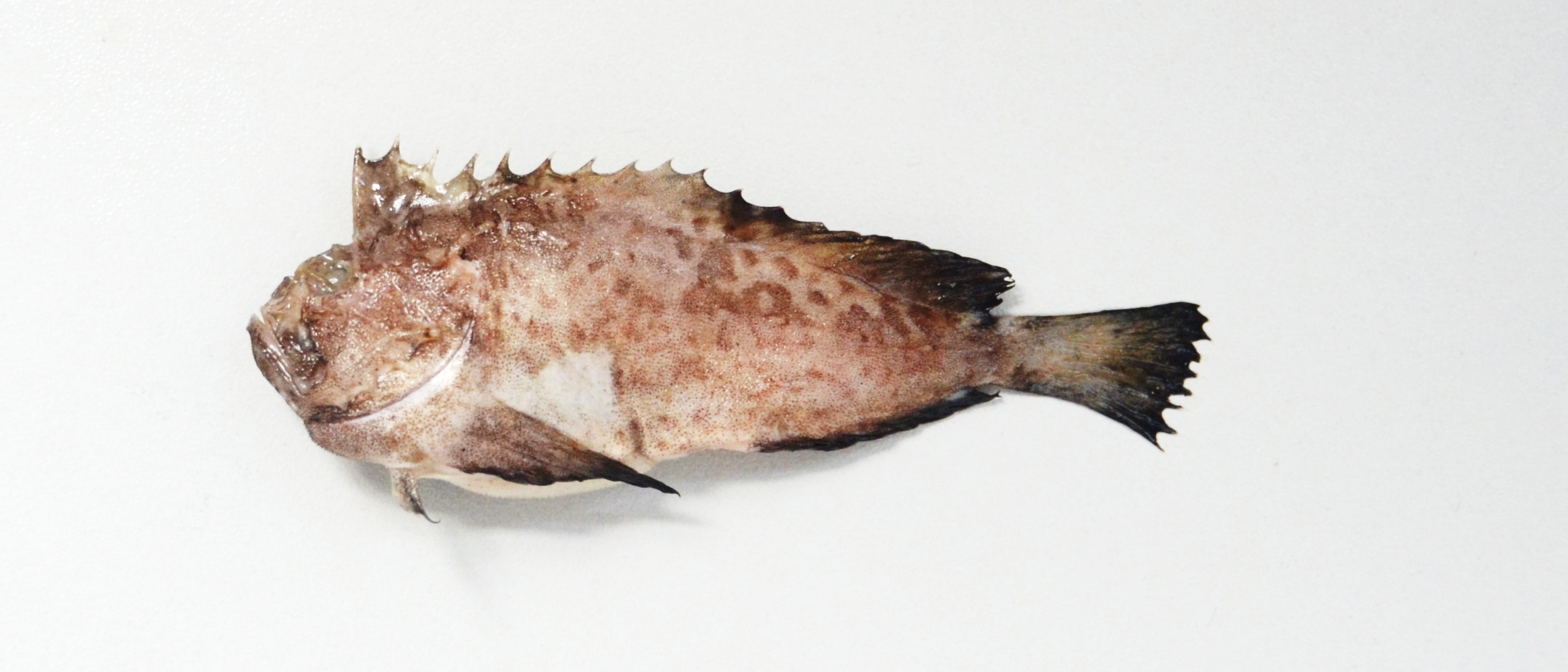 | Black scraper | *Erisphex pottii* | 11 | 77 | <http://www.fishbase.org/summary/Erisphex-pottii.html> |
| 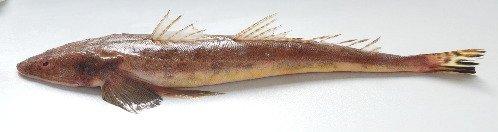 | [Bartail flathead](http://www.fishbase.org/ComNames/CommonNameSummary.php?autoctr=6056) | *Platycephalus indicus* | 14 | 143 | <http://www.fishbase.org/summary/Platycephalus-indicus.html> |


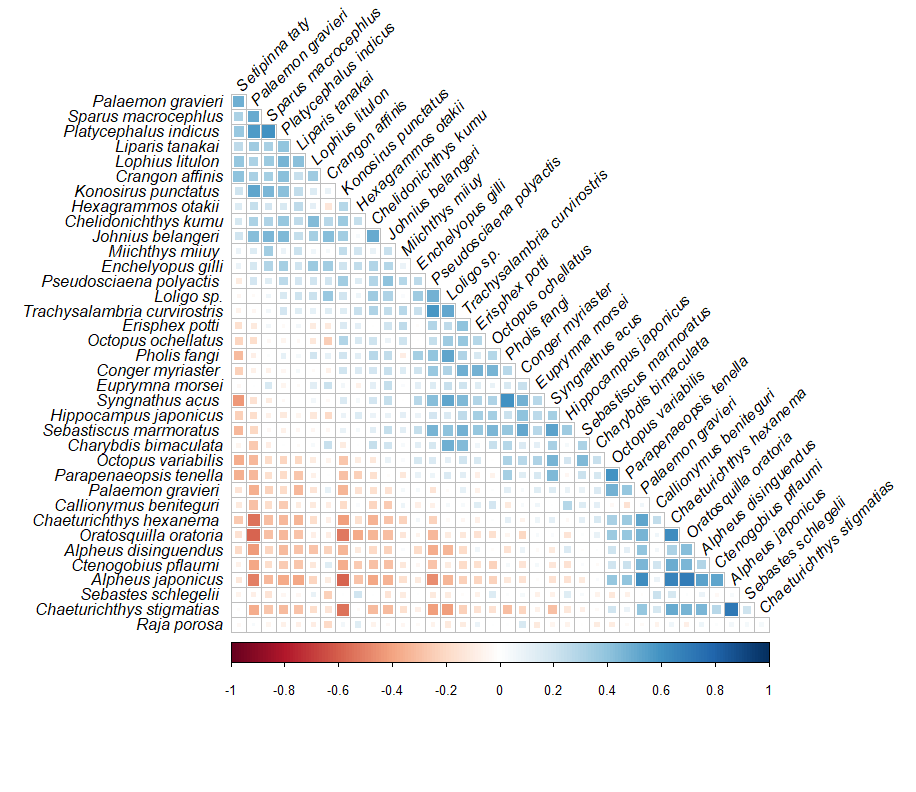


Figure S2 Species correlations in the raw data. The 37 species included six target species and 31 common species. The plot was generated from “corrplot” function in R and the species were ordered by the angular order of the eigenvectors ("AOE") for better display.

Table S2 A summary of the environmental variables and species abundance for modeling

| Variables | Min | 25% Q. | Median | Mean | 75% Q. | Max |
| --- | --- | --- | --- | --- | --- | --- |
| Longitude (E°) | 119.5 | 121.0 | 121.8 | 121.8 | 122.5 | 123.8 |
| Latitude (N°) | 35.00 | 35.75 | 36.75 | 36.78 | 37.83 | 38.50 |
| Temperature (°C) | 7.06 | 15.46 | 18.95 | 17.85 | 19.96 | 22.31 |
| Salinity (ppt) | 23.48 | 31.47 | 31.59 | 31.32 | 32.08 | 33.27 |
| Depth (m) | 7.42 | 22.76 | 30.95 | 36.32 | 47.33 | 81.29 |

| Species | Scientific names | Min | 25% Q. | Median | Mean | 75% Q. | Max |
| --- | --- | --- | --- | --- | --- | --- | --- |
| Sp1 | *Miichthys miiuy* | 0.00 | 0.00 | 0.00 | 0.05 | 0.00 | 2.50 |
| Sp2 | *Okamejei kenojei* | 0.00 | 0.00 | 0.00 | 0.07 | 0.00 | 3.51 |
| Sp3 | *Acanthopagrus schlegelii* | 0.00 | 0.00 | 0.00 | 0.08 | 0.00 | 3.28 |
| Sp4 | *Hippocampus mohnikei* | 0.00 | 0.00 | 0.00 | 0.24 | 0.00 | 3.69 |
| Sp5 | *Erisphex pottii* | 0.00 | 0.00 | 0.00 | 0.17 | 0.00 | 3.22 |
| Sp6 | *Platycephalus indicus* | 0.00 | 0.00 | 0.00 | 0.25 | 0.00 | 3.71 |
| Sp7 | *Euprymna morsei* | 0.00 | 0.00 | 0.00 | 0.72 | 0.00 | 5.86 |
| Sp8 | *Sebastiscus marmoratus* | 0.00 | 0.00 | 0.00 | 0.44 | 0.00 | 4.65 |
| Sp9 | *Palaemon gravieri* | 0.00 | 0.00 | 0.00 | 0.64 | 0.00 | 4.59 |
| Sp10 | *Conger myriaster* | 0.00 | 0.00 | 0.00 | 0.55 | 0.00 | 4.22 |
| Sp11 | *Sebastes schlegelii* | 0.00 | 0.00 | 0.00 | 0.30 | 0.37 | 3.53 |
| Sp12 | *Johnius belangeri* | 0.00 | 0.00 | 0.00 | 0.55 | 0.73 | 4.62 |
| Sp13 | *Konosirus punctatus* | 0.00 | 0.00 | 0.00 | 0.71 | 1.07 | 5.95 |
| Sp14 | *Parapenaeopsis tenella* | 0.00 | 0.00 | 0.00 | 1.40 | 3.01 | 7.58 |
| Sp15 | *Hexagrammos otakii* | 0.00 | 0.00 | 0.00 | 0.58 | 0.95 | 4.51 |
| Sp16 | *Ctenogobius pflaumi* | 0.00 | 0.00 | 0.00 | 0.85 | 1.36 | 6.53 |
| Sp17 | *Callionymus beniteguri* | 0.00 | 0.00 | 0.00 | 0.66 | 1.08 | 4.43 |
| Sp18 | *Octopus variabilis* | 0.00 | 0.00 | 0.00 | 0.66 | 1.30 | 5.23 |
| Sp19 | *Enchelyopus gilli* | 0.00 | 0.00 | 0.00 | 0.84 | 1.37 | 4.89 |
| Sp20 | *Octopus ochellatus* | 0.00 | 0.00 | 0.00 | 1.01 | 1.44 | 4.86 |
| Sp21 | *Liparis tanakai* | 0.00 | 0.00 | 0.00 | 0.64 | 1.25 | 4.59 |
| Sp22 | *Chelidonichthys kumu* | 0.00 | 0.00 | 0.00 | 0.94 | 1.83 | 5.45 |
| Sp23 | *Alpheus disinguendus* | 0.00 | 0.00 | 0.00 | 1.38 | 3.14 | 5.59 |
| Sp24 | *Syngnathus acus* | 0.00 | 0.00 | 0.00 | 1.72 | 3.29 | 7.79 |
| Sp25 | *Setipinna taty* | 0.00 | 0.00 | 0.00 | 1.38 | 2.64 | 7.57 |
| Sp26 | *Pseudosciaena polyactis* | 0.00 | 0.00 | 0.88 | 1.06 | 2.03 | 4.22 |
| Sp27 | *Trachysalambria curvirostris* | 0.00 | 0.00 | 1.52 | 2.07 | 3.75 | 8.34 |
| Sp28 | *Chaeturichthys stigmatias* | 0.00 | 0.00 | 1.28 | 1.47 | 2.56 | 4.99 |
| Sp29 | *Palaemon gravieri* | 0.00 | 0.00 | 2.65 | 2.61 | 4.88 | 7.23 |
| Sp30 | *Oratosquilla oratoria* | 0.00 | 0.00 | 1.54 | 1.75 | 2.98 | 6.54 |
| Sp31 | *Chaeturichthys hexanema* | 0.00 | 0.00 | 2.31 | 2.34 | 4.13 | 6.75 |
| Sp32 | *Alpheus japonicus* | 0.00 | 0.00 | 3.74 | 3.56 | 5.70 | 9.75 |
| Sp33 | *Pholis fangi* | 0.00 | 0.00 | 2.15 | 2.25 | 3.53 | 7.46 |
| Sp34 | *Charybdis bimaculata* | 0.00 | 0.00 | 1.90 | 2.26 | 3.68 | 7.57 |
| Sp35 | *Crangon affinis* | 0.00 | 1.84 | 4.41 | 4.14 | 6.42 | 9.62 |
| Sp36 | *Loligo sp.* | 0.00 | 1.39 | 3.22 | 3.14 | 5.00 | 7.84 |
| Sp37 | *Lophius litulon* | 0.00 | 1.13 | 1.90 | 2.01 | 2.71 | 5.08 |

Notes: Species abundance were denoted as catch data standardized by trawling speed and time, and the values were log transformed (log(x+1) in the table.


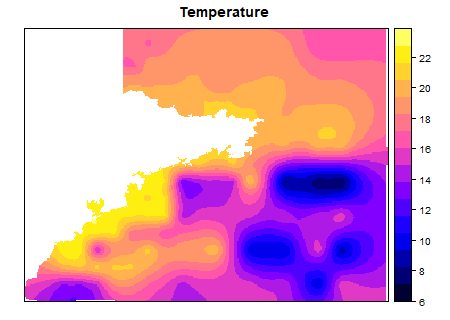

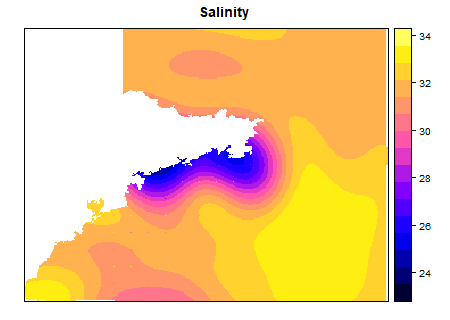


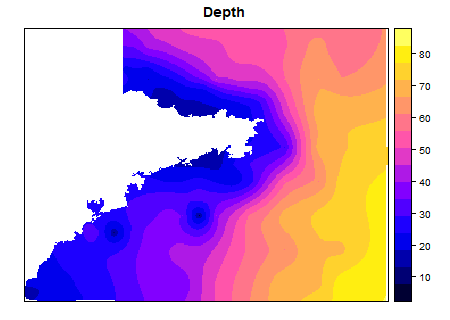


Figure S3 Maps of the environmental variables, bottom water temperate (°C), salinity (ppt), and depth (m), in the study area. A kriging method was used for interpolation from the survey data.

Table S3 Technical details of model specifications.

| Models | Specifications |
| --- | --- |
| 1. Random forest | The model was implemented using the ‘randomForest’ function in R base packages.  **#randomForest(formula, mtry=1, ntree= 1000, data= trainX, importance=T, proximity=T )**  We used ‘mtry=1’ to set the number of variables randomly sampled as candidates at each split, and used ‘ntree= 1000’ to set the number of trees. The settings were suitable for our data but might need to be tuned in other studies.  The six species were modeled with different selection of predictive variables according to our tests. A combination of temperature, depth and longitude contributed to substantially better prediction for Sp1 and Sp2; depth, longitude and latitude for Sp3 and all variables were used for other species. |
| 1. ANN | The model was implemented using the ‘nnet’ function in R base packages. It should be noted that the function can only be used to fit single-hidden-layer neural network, and an alternative “neuralnet” package may provide more flexible options.  **# nnet(formula, data= trainX , size, linout=TRUE, maxit = 2000,decay= 1e-4)**  Here size is the number of units in the hidden layer, linout=TRUE denote the output units, and decay denotes the parameter for weight decay and the small value aims to control over-fitting.  As the fitting of ANN are highly dependent on initial weighting and the structure of hidden layer for which the optimal is unknown, we generate multiple ANN with different initial weighting and hidden units (size ~ runif(6,10)) and select the model with the best fits for predictions. |
| 1. Multivariate Random Forest | The model was implemented using ‘build_forest_predict’ function in R package “MultivariateRandomForest”, in which model building and prediction were make together.  **#build_forest_predict(trainX, trainY, n_tree=500, m_feature= 2, min_leaf=5, testX)**  Here ‘n_tree’ was set to small to save computing time, m_feature is the number of randomly selected features considered for a split in each regression tree node, analog to mtry in RF, and min_leaf is the minimum number of samples in the leaf node. The two parameters need to be tuned. It should be noted that the algorithm may result in errors when trying to inverse a covariance matrix ‘solve(cov(Y))’ in the simulation, in which case the ‘ginv’ function in “MASS” package may provide a robust result. |
| 1. MANN | MANN took similar setting to ANN,  #nnet(as.matrix(trainY) ~ variables , data= trainX, size ,linout=TRUE, maxit = 1500, decay= 1e-1)  Again, multiple models were fitted and the best was used for prediction. The decay parameter should be relatively large according to our test. The number of hidden units (size) ranged from 10 to half the number of species. |
| 1. HMSC | The model was fitted using “hmsc” function in the “HMSC” package. The input data and priors of model parameters need to be formalized.  #formdata <- as.HMSCdata(trainY, trainX, Auto = coords, interceptX = TRUE)  #formpriors <- as.HMSCprior(formdata, shrinkOverall = c(100, 1), shrinkSpeed = c(20, 1))  # hmsc(formdata, family= “gaussian” , niter = 10000, nburn = 5000,thin = 10).  The parameter “Auto” was used to specify spatial-autocorrelated structure in latent variables, presented by the spatial coordinates of the samples. The model is parameterized using MCMC and use the sparse infinite factor prior distribution to select the proper number of latent variables. Large values of shrinkage parameters ‘shrinkOverall’ and ‘shrinkSpeed’ was used to control the number of autocorrelated latent variable. Quadratic terms of temperature and depth were added to the model to accommodate nonlinear responses. |
| 1. Gjam | The model were fitted using the “gjam” function in “gjam” package.  **#ml= list(ng = 10000, burnin = 1000, typeNames = "CA")**  **#gjam(formula, ydata= trainY , xdata= trainX, modelList =ml ).**  The model uses a censoring process to integrate discrete and continuous data on the observed scales. The dimension reduction scheme is conducted using a low-order approximation of species covariance matrix. The predictor variables should be explicitly included in the function, and the type of the model were specified in a “modelList” parameter, in which “CA” represented continuous abundance data and a range of other “typeNames” were available. Quadratic terms of temperature and depth were also included in this model. |


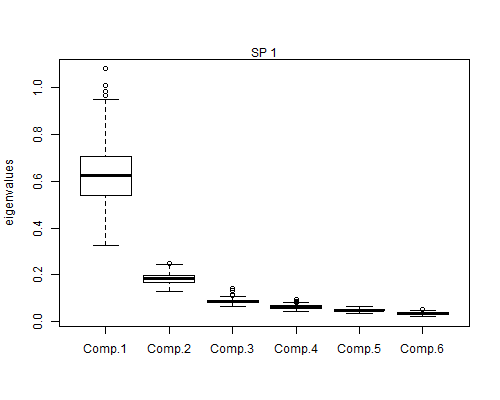

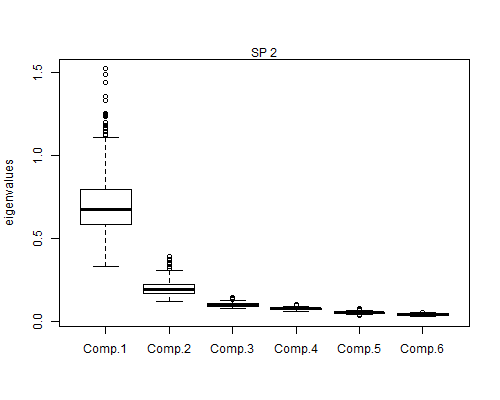


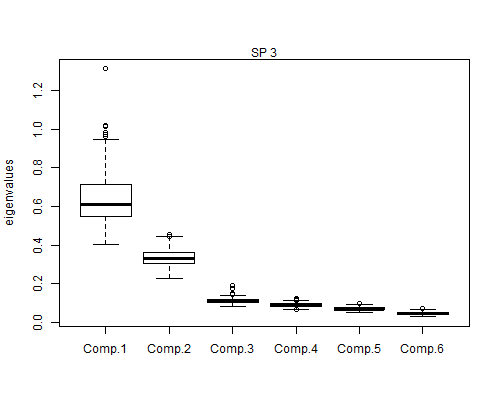

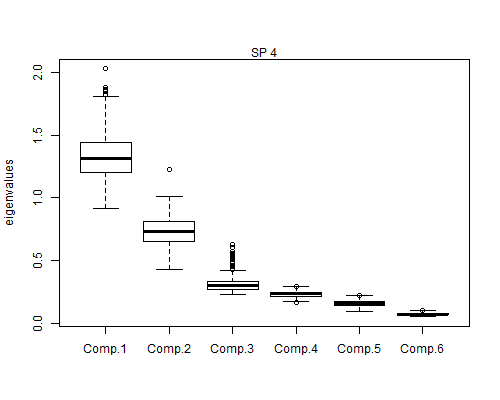


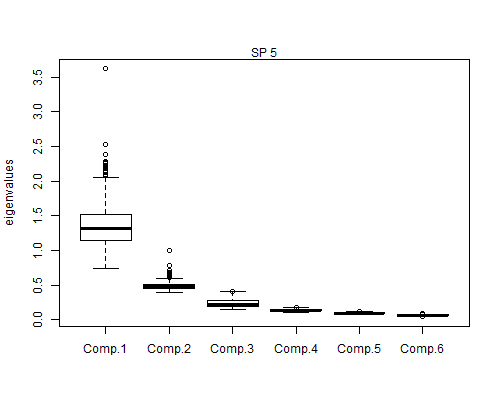

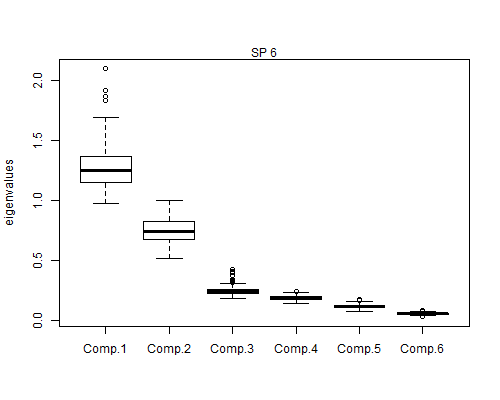


Figure S4 Principal component analyses on the prediction of six different models, RF, ANN, MRF, MANN,HMSC and GJAM for six targeted rare species. X-axis denotes the resultant principal components and Y-axis denoted the corresponding eigenvalues in multiple simulations. A large eigenvalue in the first component implied high correlations among the results from different models.


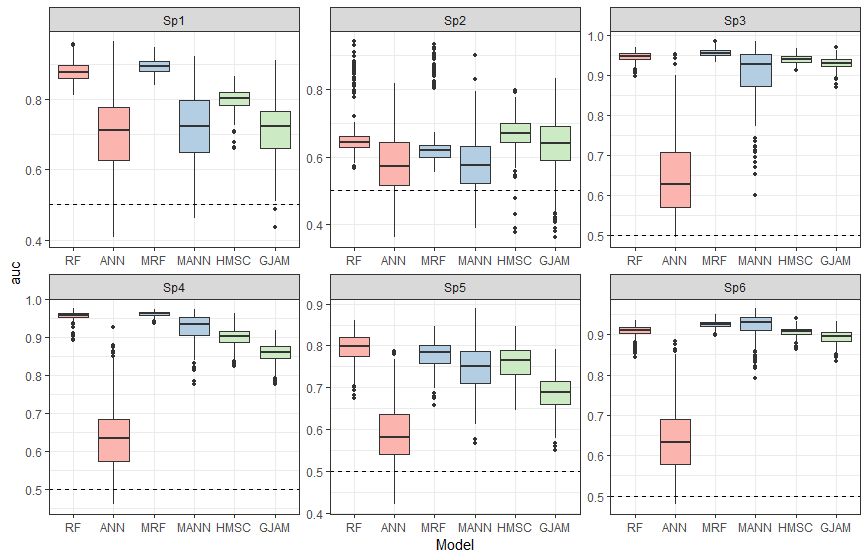


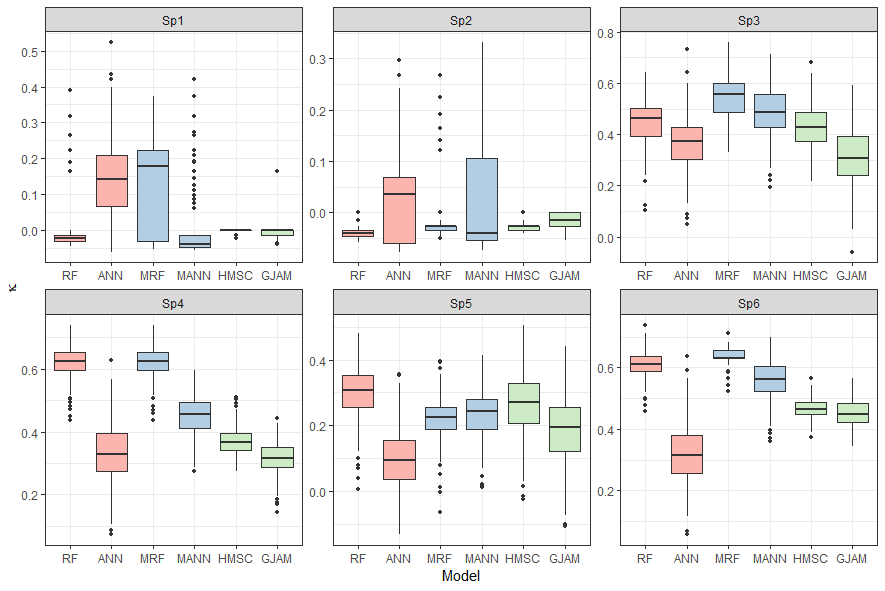


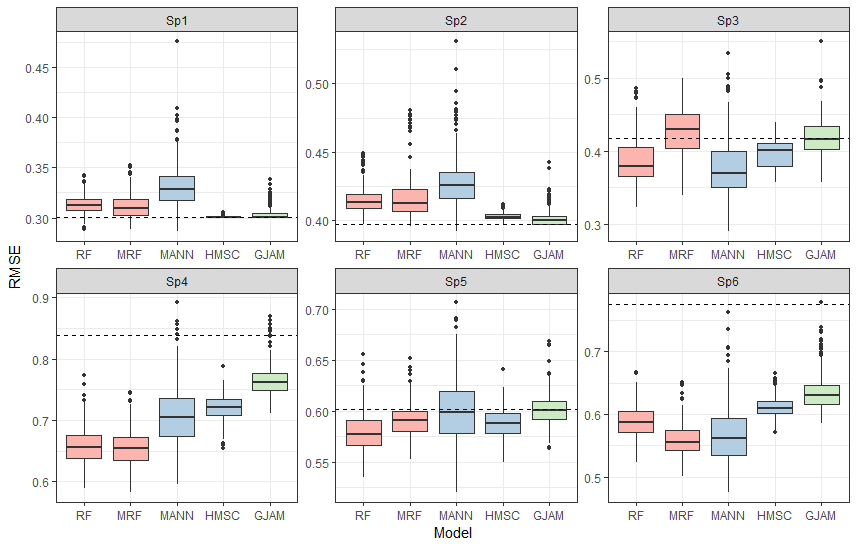


Figure S5 Predicting performances of SDMs on the distribution of six target species. The prediction of occurrence was evaluated by the area under the curve of receiver operating characteristic (auc) and Cohen’s coefficient (κ), and prediction of abundance was evaluated by partial relative bias of non-zero data (PRB) and rooted mean square error (RMSE). The dash line in the last plot denoted a baseline of RMSE derived from all-zero predictions. The results were supplements to Figure 1 in the main texts.


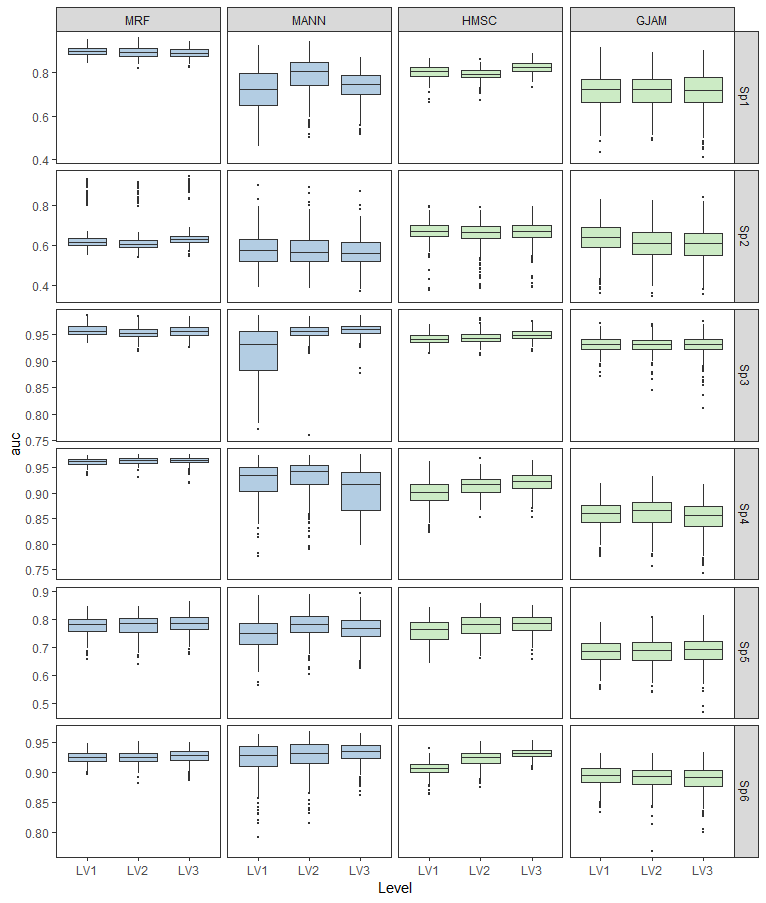


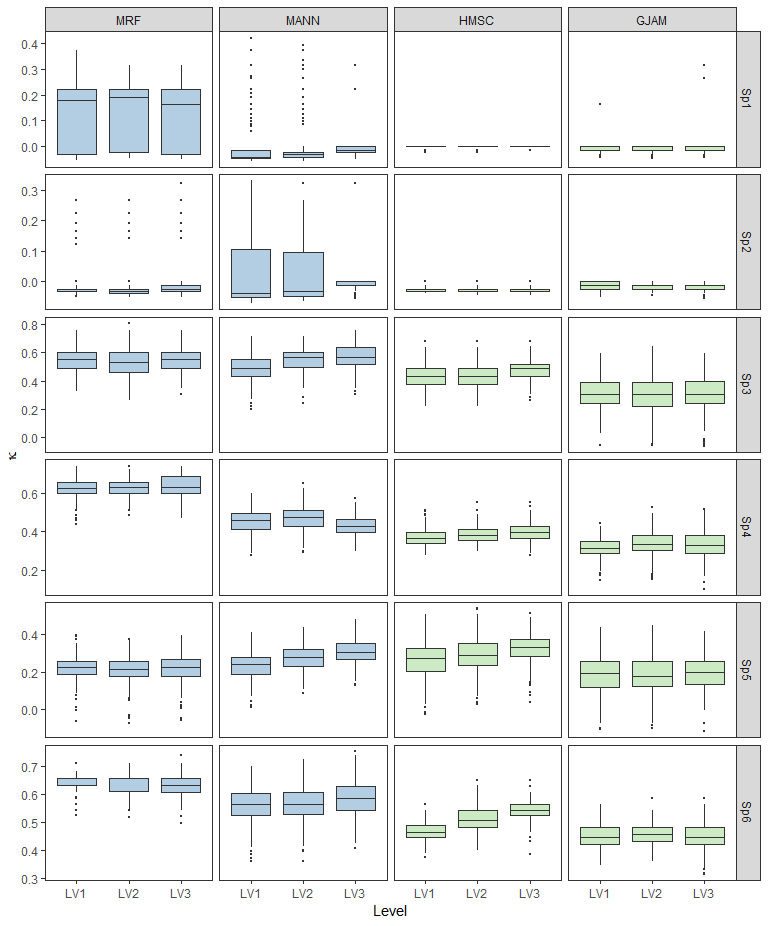


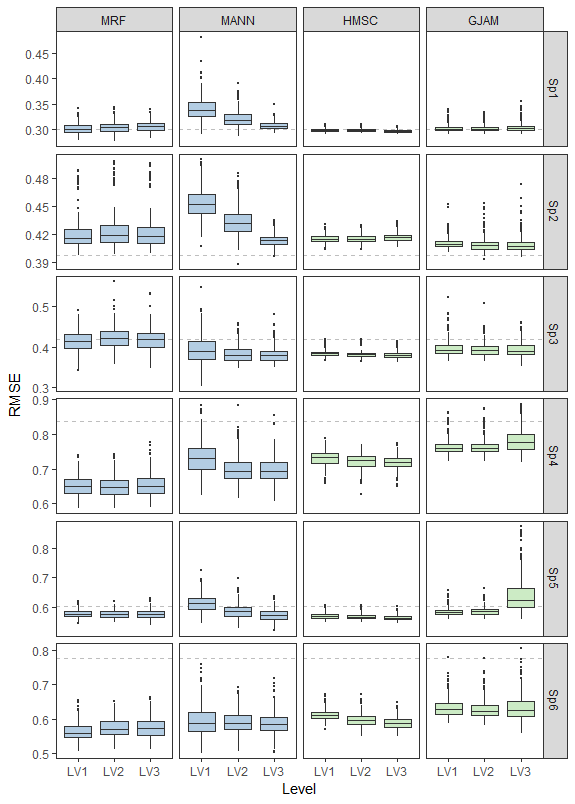


Figure S6 The influences of the number of ancillary species on the predictive performance of JSDMs. The levels in the X-axis denoted different thresholds of species correlation to select ancillary species. The figures were supplements for Figure 2 in the main tests.


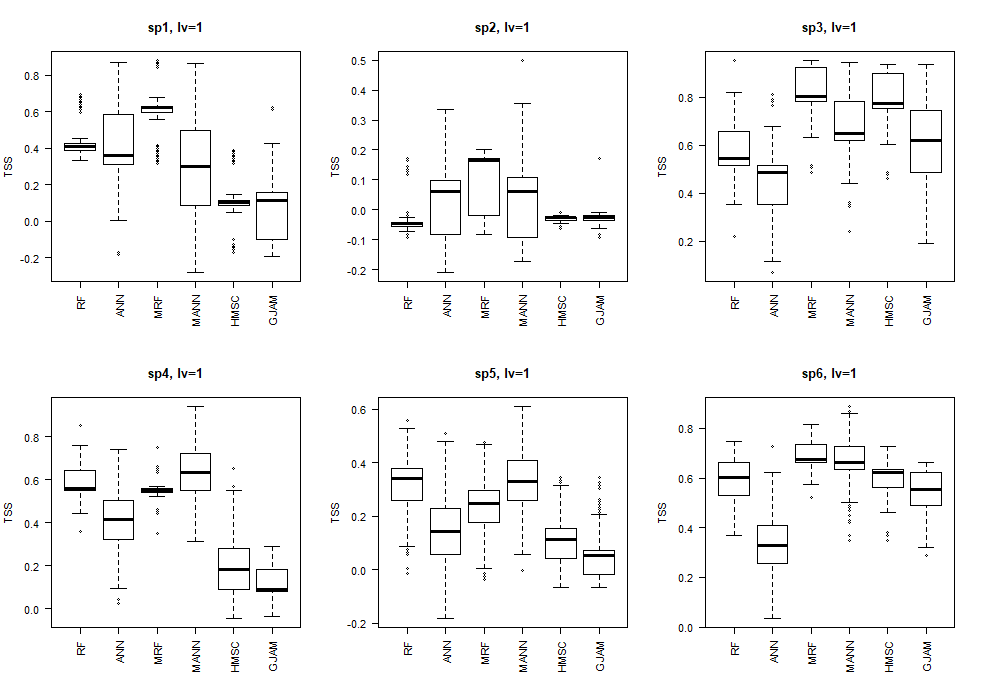


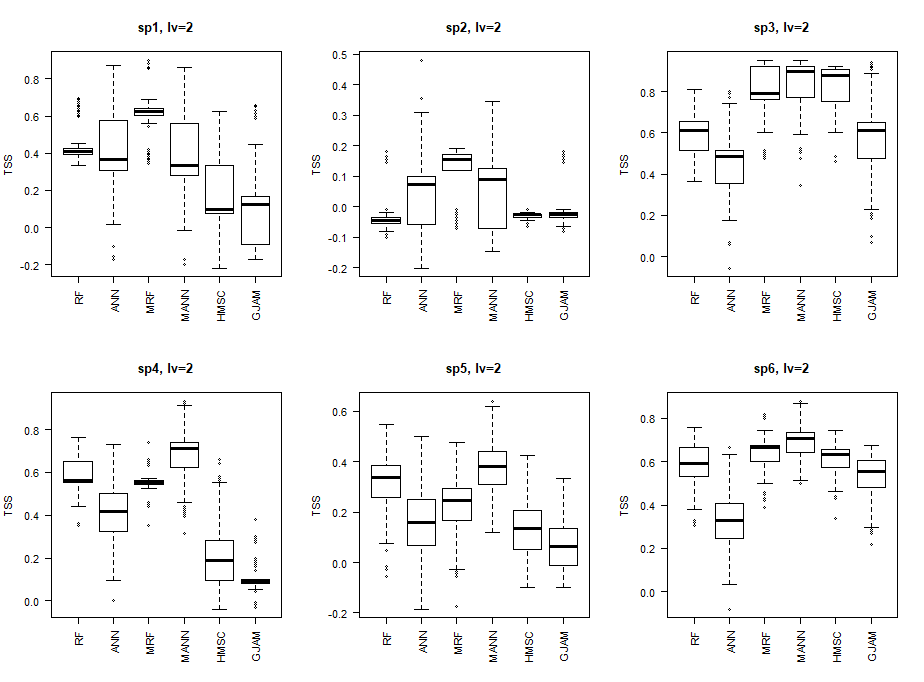


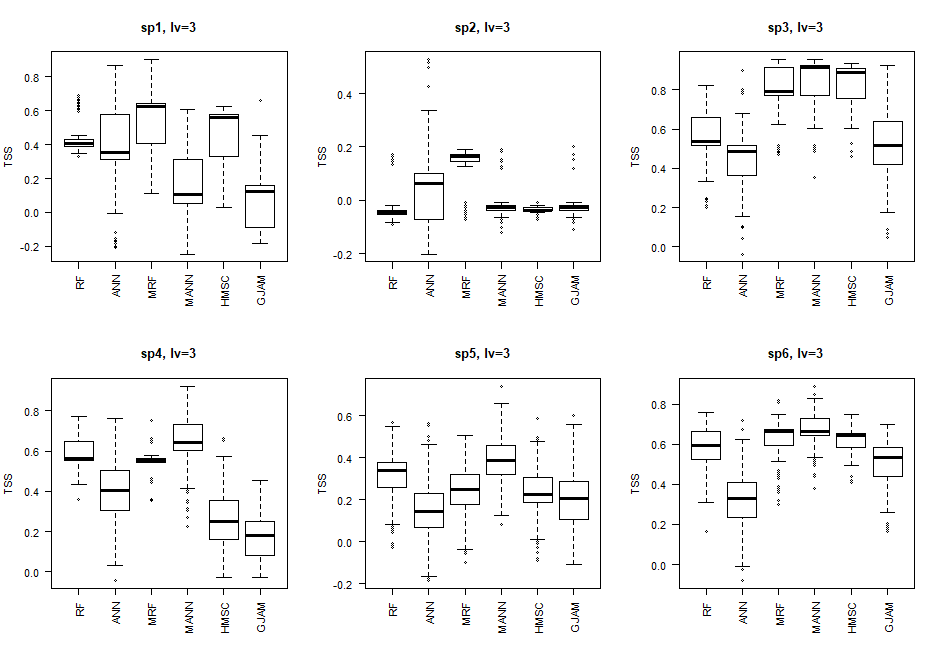


Figure S7 Predicting performances of SDMs on the distribution of six target species measured by True Skill Statistic (TSS). The lv of each plot indicated the levels of species filtering.


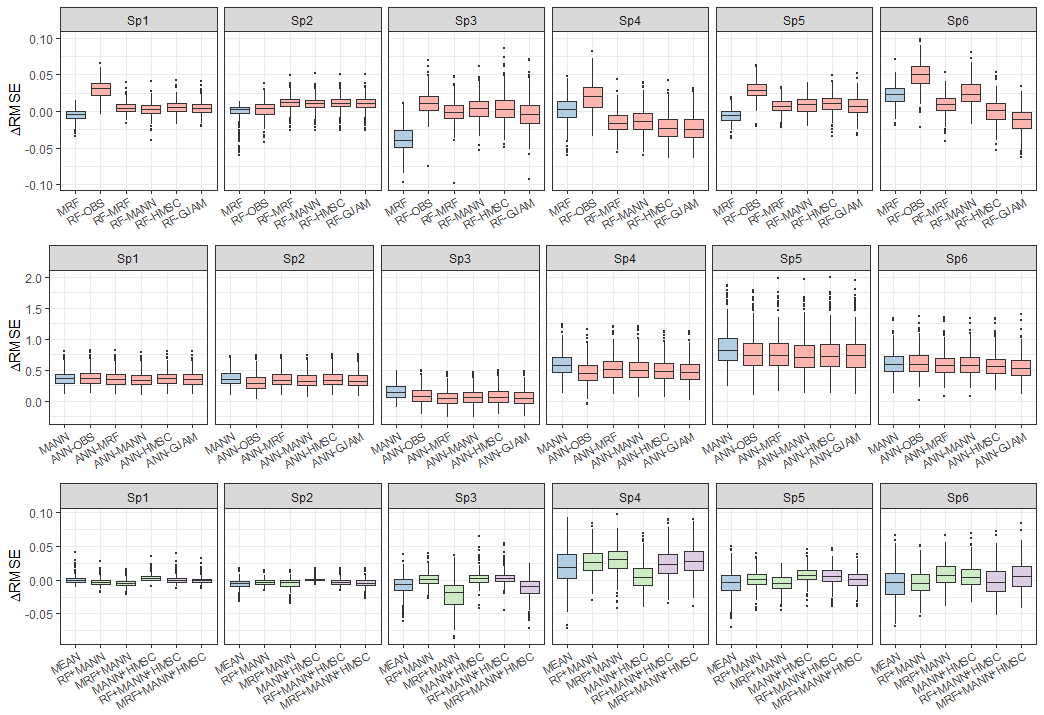


Figure S8 The effects of conditional prediction on improving predictive performances for rare species. The ΔRMSE in the first two rows indicated the decreases of RMSE in conditional models compared to that of the RF and ANN with single species, respectively. RF-OBS and ANN-OBS denoted the predictions conditioning on real observations (survey data), and others were conditional on the prediction of JSDMs, respectively.
